# Editorial: Mental health promotion and suicide prevention in a changing world

**DOI:** 10.3389/fpsyt.2022.1129979

**Published:** 2023-01-17

**Authors:** Qing Zhao

**Affiliations:** ^1^CAS Key Laboratory of Mental Health, Institute of Psychology, Chinese Academy of Sciences, Beijing, China; ^2^Department of Psychology, University of Chinese Academy of Sciences, Beijing, China

**Keywords:** mental health promotion, suicide prevention, COVID-19, mental vulnerability, self-esteem

*Mental Health Promotion and Suicide Prevention in a Changing World* (July 2021 to June 2022) was a special research project conducted during the global spread of COVID-19. Our project editors were concerned that individuals' mental wellness could be vulnerable due to the pandemic and other concurrent natural or man-made disasters (e.g., earthquakes and regional conflicts). Moreover, bereavement, unemployment, and economic recession due to these disasters could further build up emotional stress upon the public. Meanwhile, pandemic regulations administered in each community (e.g., lockdowns and social distancing measures) might further undermine social support. Consequently, the risk of suicidal ideation and behavior during these changing times could be concerning. This project aims to follow the above research concerns. In total, 14 studies were published *via* this research project, with dedications by 103 authors, 26 reviewers, and five editors. Five essential research questions were discussed among these researchers:

**WHOM** should we be concerned about?

In this project, researchers investigated mental health-related issues with people from aboriginal communities in Aotearoa/New Zealand (e.g., the Māori people; Pavlova et al.), Asian cultures (e.g., Indians; Ramesh et al.), and Western countries (e.g., Germans; Kohls et al.). Participants of this project covered children (e.g., Kohls et al.), adolescents (e.g., Li, Zhan et al.), young adults (e.g., Huang et al.), low-income cohorts (e.g., Kaniuka et al.), police officers (Hofmann et al.), and suicide prevention professionals (e.g., Roškar et al.). As Roškar et al. highlighted, even the professional knowledge of these suicide prevention professionals would not make them “immune” to mental illnesses. Ergo, mental health promotion and suicide prevention should be considered a global project, covering the welfare of a broad spectrum of populations.

**WHAT** are the risk factors for mental health issues and suicide attempts?

According to Dat et al. and Li, Zhan et al., people were more likely to be trapped by suicidal ideation if they labeled themselves as “unimportant,” “unpromising,” and “disconnected.” Moreover, Li, Zhan et al. emphasized that “unimportant” is the central note linking adolescents' negative concepts of abuse, depression, and suicidal ideation. Similarly, problematic drinking (Kaniuka et al.) and financial hardship (Mathieu et al.) could threaten self-esteem, decrease resilience, and increase mental health concerns. In contrast, self-esteem enhancement activities can be the “antidote” (Dat et al.; Rudd et al.). Some “antidotes” prescribed by the current researchers were empowerment-oriented intervention (Park et al.), self-efficacy promoting game playing (Li, Zheng et al.), and active coping strategy learning (Kaniuka et al.).

**HOW** did COVID-19 impact the public's mental wellness?

Researchers considered that suicidal behavior and ideation during COVID-19 might not be solely attributed to the pandemic *per se* (Mathieu et al.; Clapperton et al.). In contrast, researchers found that the spikes in helpline calls and in suicidal risks corresponded to the consequent events of the pandemic, such as lockdowns and economic recession (Mathieu et al.; Pavlova et al.). These consequent events might increase individuals' feelings of isolation and decrease their self-assurance when facing life problems (Mathieu et al.).

Moreover, COVID-19's impact on the suicide rate is also impacted by people's age, sex, culture, and other demographic characteristics. Clapperton et al. found that the suicide rate of young male Westerners (e.g., Australians) increased during the pandemic. Similarly, the demand for helpline services was enhanced among youth and cohorts with financial issues in Aotearoa/New Zealand (Pavlova et al.). In contrast, Ramesh et al. mentioned that antecedent factors for suicide during COVID-19 could be inconsistent for Indian males (i.e., due to financial issues, such as unemployment) and females (i.e., due to interpersonal and affective issues, such as domestic violence and depression). Referencing World Health Organization's report [(1), p. 10], the male/female sex ratio of suicide rates tended to be larger in Western (e.g., America and Russia) than in Asian countries (e.g., China and India). The above findings hinted at a “culture–sex interaction effect” on suicide, which is worth attention by future researchers.^1^

**WHAT** can we do now?

Researchers proposed that playing simple and manageable music video games could help youths with depression (Li, Zheng et al.). Similar activities may help people re-establish self-efficacy (Li, Zheng et al.). Self-efficacy and self-esteem, in turn, form a “bubble” protecting people from mental health issues (Dat et al.). In contrast, time-wasting electronic activities (e.g., excessive smartphone use) could induce self-blame and weaken the “bubble” (Huang et al.).

Meanwhile, telephone-based and messenger-based counseling services are necessary for mental health promotion, especially during the pandemic (e.g., Pavlova et al.). Notably, females were more likely to express their suicidal ideation and attempts (Kohls et al.), whereas the suicide rate of males was significantly higher (6). Shi et al. (6) discussed that males might conceal their mental vulnerability to protect their masculine pride; this caused their mental health issues to be underdiagnosed and undertreated. Similarly, researchers remarked that mental health promotion should be adapted to clients' cultures (e.g., the Māoris had a gradually decreased demand for helpline services during COVID-19; Pavlova et al.). Helpline workers and other psychological professionals should be aware of the above sex and cultural differences.

Furthermore, researchers pointed out that formal education about suicide prevention (Hofmann et al.), mental health stigma reduction (Roškar et al.), child protection (Li, Zhan et al.), and firearm safety plans (Rudd et al.) are all necessary for achieving the goal of mental health promotion and suicide prevention in our society. Moreover, these educational modules should be provided to high-risk populations, suicidologists, police officers, and other relevant professionals (Hofmann et al.; Park et al.; Roškar et al.). Finally, the current researchers also highlighted that social welfare (e.g., minimum wage and unemployment benefits) in tandem with emotional support from families and friends could be the last defense for people considering suicide (Mathieu et al.; Pavlova et al.; Huang et al.).

**WHAT** shall we consider for the future?

As reflected by this project, our current knowledge about suicidal ideation and behavior was mainly based on registered data and self-report studies. In contrast, the possible biomarkers of suicidal behavior and the neurological networks underpinning suicidal ideation were largely unknown. As Dat et al. stressed, every suicide could negatively affect 6–135 people [also see (7)]. A better understanding of the biomarkers and neurological networks would help us to predict people's suicide with higher accuracy and present more timely and solid suicide prevention and mental health support to those high-risk individuals and people around them.

## Author contributions

QZ wrote the editorial.

## Footnotes

^1^To date, researchers have observed the “culture–sex interaction effect” in studies of self-esteem (2), emotional processing (3), and interpersonal empathy (4, 5).

## References

[1] World Health Organization. Suicide in the World: Global Health Estimates. (2019).

[2] Schmitt DP. The evolution of culturally-variable sex differences: Men and women are not always different, but when they are… it appears not to result from patriarchy or sex role socialization. Evolution Sexual. (2015) 2015:221–56. 10.1007/978-3-319-09384-0_11

[3] Pugh ZH Huang J Leshin J Lindquist KA Nam CS. Culture and gender modulate dlPFC integration in the emotional brain: Evidence from dynamic causal modeling. Cognit Neurodyn. (2022) 2016:1–16. 10.1007/s11571-022-09805-2 PMC9871122 36704624

[4] Zhao Q Neumann DL Cao Y Baron-Cohen S Yan C. Culture–sex interaction and the self-report empathy in Australians and Mainland Chinese. Front Psychol. (2019) 10:396. 10.3389/fpsyg.2019.00396 30914986 PMC6422933

[5] Zhao Q Zhou L Ren Q Lu X Sun X Neumann DL. Culture–sex interaction in self-report empathy: The theory and meta-analyses. PsyCh J. (2022). 10.1002/pchj.598 36104300

[6] Shi P Yang A Zhao Q Chen Z Ren X Dai Q. A hypothesis of gender differences in self-reporting symptom of depression: Implications to solve under-diagnosis and under-treatment of depression in males. Front Psychiatry. (2021) 12:e589687. 10.3389/fpsyt.2021.589687 34759845 PMC8572815

[7] Cerel J Brown MM Maple M Singleton M Van de Venne J Moore M. How many people are exposed to suicide? Not six. Suicide Life-Threat Behav. (2019) 49:529–34. 10.1111/sltb.12450 29512876

